# Evaluating the Effect of Dietary Protein–Energy Ratios on Yak Intestinal Microbiota Using High-Throughput 16S rRNA Gene Sequencing

**DOI:** 10.3390/vetsci12030208

**Published:** 2025-03-01

**Authors:** Yanbin Zhu, Yangji Cidan, Munwar Ali, Sijia Lu, Usama Javed, Zhuoma Cisang, Deji Gusang, Quzha Danzeng, Kun Li, Wangdui Basang

**Affiliations:** 1 Institute of Animal Husbandry and Veterinary Medicine, Tibet Academy of Agriculture and Animal Husbandry Sciences, Lhasa 850009, China; zhuyanbin126@126.com (Y.Z.); 13889092363@163.com (Y.C.);; 2 College of Veterinary Medicine, Nanjing Agricultural University, Nanjing 210095, China; drmunwarali06@gmail.com (M.A.);

**Keywords:** protein–energy ratio, 16S rRNA sequencing, microbial diversity, yak intestinal microbiota

## Abstract

This study investigated the impact of varying dietary protein–energy ratios on the intestinal microbiota composition in postpartum weaned female yak. For this study, forty yaks were divided into four groups and provided with different dietary treatments (group FA: high-energy high-protein, FB: high-energy low-protein, FC: low-energy high-protein, and FD: control group, provided with 48% alfalfa hay, 48% oat grass, and 4% premix) to investigate the variations in microflora profiles and metabolic responses. Rectal fecal samples (n = 24 × 2) were collected at day 15 and 30, from all four groups, and total DNA was extracted to estimate microbial heterogeneity and community structures by 16S rRNA sequencing focusing V3–V4 regions, using the Illumina Nova Seq 6000 platform. The results revealed a total of 5,669,645 raw data sequences (3,189,115 and 2,480,530 from day 15 and day 30, respectively). Results showed that groups FA and FB had enhanced protein metabolism and microbial diversity, which was marked by a significant increase (*p* < 0.05) in abundance of *Ruminococcus*. Conversely, the FD group showed a low level of microbial diversity with a significant (*p* < 0.05) predominance of *Clostridium* and Proteobacteria, indicating microbial dysbiosis and metabolic stress. It was concluded that imbalanced diets (groups FC and FD) upregulated the stress-related pathways with no favorable microbial shifts, whereas, dietary treatments in group FA and FB significantly (*p* < 0.05) supported the pathways involved in amino acids and carbohydrate metabolism and beneficially shifted the gut microbiota. These findings emphasize the importance of postpartum supplementation with appropriate proportions of protein and energy feed to promote optimal microbial health and metabolic functioning, particularly for yaks inhabiting high-altitude regions, which is a challenging environment.

## Simple Summary

Yaks, which are essential for milk, meat, and transportation in Tibet’s high-altitude areas, rely on healthy digestion to thrive in challenging environments. This study explored how different diets, varying in protein and energy proportions, affect the gut bacteria of postpartum female yaks. Forty yaks were divided into four groups: three were given specially formulated feeds with high or low protein–energy ratios, while the control group ate a standard diet. Fresh fecal samples were analyzed to identify gut bacteria changes. Results showed that yaks fed with high-protein diets had more diverse gut microbes, including beneficial bacteria like *Ruminococcus*, which aids digestion and nutrient absorption. In contrast, the control group had less diverse gut bacteria and increased harmful microbes. Balanced protein–energy diets improved metabolic functions, reduced stress-related bacteria, and supported efficient energy use. These findings highlight the importance of tailored feeding strategies for postpartum yaks to maintain gut health, enhance recovery, and boost productivity. Adoption of these diets could mean healthier herds, better reproductive success, and improved livelihoods for local herders in challenging environments, offering practical guidance to optimize yak nutrition, ensuring sustainable farming and economic resilience for high-altitude communities.

## 1. Introduction

The gut microbiome plays a crucial role in improving the health and performance of animals, especially in ruminants like yaks [1]. The term “gut flora” refers to the divergent community of microorganisms that includes bacteria, archaea, fungi, and viruses, which are the main inhabitants of the digestive tract in animals and humans [2]. These microbes and their metabolic products produce an environment in the gut that enhances metabolism, nutrition absorption, immune modulation, and fermentation efficiency in ruminants, leading to improved animal health [3,4]. Contemporary studies have further highlighted the gut microbiota’s role in regulating immune responses, including recognizing beneficial versus harmful pathogens [3]. However, microbial community dysbiosis within the gut can lead to digestive and metabolic disorders, resulting in low animal productivity [5]. Therefore, understanding the gut microbial community’s structure and functions is essential for improving livestock health, enhancing feed efficiency, and developing microbiome-targeted interventions to prevent diseases.

Studies have shown that perinatal nutritional supplementation improves reproductive performance in yaks by reducing body weight loss and improving metabolic adaptation [6]. Supplementation and early weaning can alleviate negative energy balance and reduce lipid mobilization during lactation [6]. Studies show that yaks receiving nutritional supplementation had higher body weight gain, serum glucose, globulin, and total protein concentrations [6]. Significant research has been conducted on the gut microflora of livestock animals such as ruminants. However, studies focusing specifically on the yak’s gut microbiome in response to dietary treatments, particularly in lower gastrointestinal tract sections, remain limited [7].

Yak (*Bos grunniens*) is a herbivorous species like cattle and buffalo, and it is mostly found in the Qinghai–Tibetan Plateau Area (QTPA), providing a main source of milk, meat, and transport to the local population [8,9]. The recovery speed of female yaks after weaning determines the reproductive efficiency of the entire herd, indirectly affecting the economic benefits of the yak breeding industry. Furthermore, as yaks live in high-altitude environments and graze on forage with poor nutritional value, their gut microfloral communities are more evolved, allowing them to effectively extract energy from their limited resources. While several studies have explored the yak gut microbiota, they have focused on the rumen microbiome or have limited sampling to one or two specific regions [10]. This limited approach does not fully capture the diversity of microbial communities, as environmental factors significantly influence these populations. Thus, further research on the makeup of the intestinal microbiome, particularly in the lower gastrointestinal tract (GIT), is essential. Additionally, limited data exist on how the yak’s intestinal microbiota adapt to changing environmental and physiological conditions, and there is a need to explore the gut microbiota’s roles in immune modulation, disease prevention, and energy metabolism, which could guide strategies to enhance yak health and productivity [10].

The emergence of 16S rRNA sequencing technology has transformed microbiota research by empowering the identification of microbial communities, especially novel bacteria, and their isolates. This technology is useful for rare, uncultivable, slow-growing bacteria with unusual phenotypic profiles [11]. The 16S rRNA sequence, coding for the ribosomal 16S rRNA, contains both conserved and hypervariable regions; these features enable reliable phylogenetic and taxonomic identification at the species level, making it a powerful tool for bacterial analysis [12,13,14]. Traditional culture-based methods for microbial profiling are limited as they can only grow and analyze easily cultivable organisms. In contrast, modern sequencing techniques, such as Illumina-based 16S rRNA sequencing, generate extensive data that allow for accurate assessment of microbial diversity [15]. This shift has been accompanied by the development of bioinformatics tools, including Quantitative Insights into Microbial Ecology (QIIME 2) and Divisive Amplicon Denoising Algorithm 2 (DADA2), which facilitate detailed analyses of microbial diversity at high resolution, including alpha and beta diversity metrics [16]. This progress has made the study of microbial ecology more systematic and economical.

This study uniquely explores the impact of specific dietary protein–energy ratios on the intestinal microbiota of postpartum weaned yaks, an understudied yet critical phase in high-altitude livestock management. Departing from previous research focused on rumen microbiota or single-region sampling, this work comprehensively analyzes microbial shifts in the lower gastrointestinal tract using 16S rRNA sequencing, integrating functional pathway predictions with serum biochemistry to elucidate diet–microbiome–metabolism interactions. The formulation of experimental diets based on locally relevant feed components and their influence on microbial biomarkers offers unique insights into optimizing nutrient utilization in extreme environments, providing yak breeders with actionable strategies to enhance postpartum recovery and reproductive efficiency through balanced protein–energy supplementation, potentially mitigating economic losses in high-altitude regions by promoting microbial resilience and nutrient absorption; future research should explore longitudinal effects on yak productivity and investigate microbiome-targeted interventions to amplify beneficial microbial taxa, expanding this framework to other plateau-adapted livestock to advance sustainable agriculture in challenging ecosystems [17,18,19,20,21,22,23,24].

## 2. Materials and Methods

### 2.1. Animal Experiments, Sample Collection, and Preparation

This experiment was conducted in Linzhou Jingmu Agricultural and Animal Husbandry Development Company (29°58′52″ N, 91°16′47″ E) in Lhundup, Lhasa City, Tibet Autonomous Region. Forty female yaks in the experiment (4–6 months postpartum) were isolated with their calves. The sample size was determined based on prior studies [25,26]. These female yaks were randomly divided into four groups (10 yaks per group: a control group and three test groups). Each group was placed in different enclosures and had free access to drinking water. Three types of feed with various protein and energy ratios were prepared based on the nutritional requirements of postpartum weaned yaks. The specific feed formulas are shown in Table 1, while the nutrient composition of the forage is shown in Table 2. The control group (group FD) was fed with ordinary grass (48% alfalfa hay and 48% oat grass) and 4% premix, while the three different formulations of feed were given to three different experimental groups (group FA: high-energy high-protein, FB: high-energy low-protein, and FC: low-energy high-protein). The forty yaks were fed with pre-prepared feed at 9:00 am and 3:00 pm every day. The total experimental period consisted of 30 days and samples were collected on day 15 and 30.

In the current study, all forty-eight (n = 24 × 2) rectal content samples were collected from yaks in two experimental stages (on day 15 and 30) to evaluate the impact of different dietary protein–energy ratios on their intestinal microflora composition. Each sample was obtained aseptically from individual yaks. All collected samples were preserved on dry ice and brought to the research laboratory, where they were conserved at −80 °C until the DNA was extracted for future use.

### 2.2. Ethical Statement

All procedures performed in this research were approved by the Laboratory Animal Welfare and Ethics Committee of the Institute of Animal Husbandry and Veterinary Medicine, Tibet Academy of Agriculture and Animal Husbandry Science, and the ethics committee of Nanjing Agricultural University (NJAU.No20220305025). All methods were carried out by relevant guidelines and regulations.

### 2.3. Experimental Process (Workflow)

The whole high-quality microbial DNA genome was extracted from samples with the help of OMEGA Mag-bind Soil DNA Kit (Omega Bio-tek, Inc., Norcross, GA, USA). The purity of the extracted DNA was checked using a spectrophotometer (Thermo Scientific, Waltham, MA, USA), and microbial profiling was conducted by expanding the 16S rRNA gene’s hypervariable sections (V3–V4) [27]. Hypervariable regions (V3/V4) were amplified by specific bacterial 16S rRNA gene primers: the forward primer (5′-ACTCCTACGGGAGGCAGCA-3′) and the reverse primer (5′-GGACTACHVGGGTWTCTAAT-3′), with high-fidelity DNA polymerase [28,29] provided by Beijing Quanshijin Biotechnology Co., Ltd., Beijing, China. Each sample was identified using specific primers with barcode sequences used in PCR amplification. The PCR reaction mixture for each amplification reaction consisted of 12.5 μL PCR Mix, 1.0 μL DNA, 1.0 μL of each forward and reverse primers, and 9.5 μL dd.H_2_O, with a total reaction volume of 25 μL. PCR amplification consisted of a total of 35 PCR cycles, with each cycle having an initial hot start pre-denaturation temperature of 95 °C (5 m), then 95 °C (15 s); the primer annealing temperature Tm was 50 °C (15 s), the elongation was 72 °C (45 s), which was followed by extension at 72 °C (10 m), and finally storage at 4 °C. After performing PCR, the resultant amplified PCR products were assessed using 2% agarose gel electrophoresis. PCR products were then purified and recovered with magnetic beads (Vazyme VAHTSTM DNA Clean Beads).

Additionally, the reactions were conducted using high-fidelity DNA polymerase for accuracy and to point out minor errors. The amplified products were verified with the help of 2% agarose gel electrophoresis. To improve the precision and consistency of the data scrutiny results, filtration of raw data was performed using QIIME 2′s default parameters. This process included noise reduction and quality control measures, such as splicing and chimerism detection, which involved filtering out sequences with an abundance of less than 10 across all samples. The resultant amplicon sequence variants (ASVs) were evaluated with the help of multiple diversity indicators and sequencing extent detection. Additionally, statistical assessments of community structures were performed at various taxonomical ranks.

After that, the Quant-iT PicoGreen dsDNA Assay Kit (Thermo Fisher Scientific, Waltham, MA, USA) was used to cut and upgrade the target sections, and quantification of purified products was performed by using a BioTek FLx800 Microplate Reader (BioTek Instruments, Winooski, VT, USA), which had fluorescent dye to measure DNA concentration [30,31,32] accurately. Moreover, an archive of sequencing was generated with the help of the TruSeq Nano DNA LT Library Prep Kit (from Illumina), a superior quality check was ensured by using the Agilent Bioanalyzer 2100 (Agilent Technologies, Santa Clara, CA, USA) and the Promega QuantiFluor system (Promega Corporation, Madison, WI, USA) was used to check unification and number of libraries. Based on phylogenetic information, Unifrac was used to measure the distance between sets of taxa by applying it to 16S rRNA libraries. The libraries with high-quality standards were selected to be exposed to paired-end sequencing on the Illumina NovaSeq 6000 platform (Bioyi Biotechnology Co., Ltd., Wuhan, China) [33].

### 2.4. Bioinformatics and Data Analysis

After sequencing was completed, the primary data were acquired in FASTQ format. Furthermore, QIIME 2 software (Version: 2024.10) engaged in the preprocessing of paired-end reads, including quality filtering, merging paired-end reads, and removing the adapter. The DADA2 algorithm was used to denoise, generate ASVs, and remove chimera. The Silva 138 database (16s/18s rDNA) and the classify-sklearn module in QIIME 2 were used to explain these ASV sequences taxonomically. Additionally, taxonomic classification was also conducted at phylum, genus, and species levels. To analyze within-sample diversity or alpha diversity, indicators such as Simpson, Shannon, Chao1, and observed species were monitored. For between-sample diversity, or beta diversity, principal component analysis (PCA), principal coordinate analysis (PCoA), and non-metric multidimensional scaling (NMDS) analyses were conducted, relying on both weighted and unweighted UniFrac distances, as well as the Bray–Curtis distance metric [34,35]. Rarefaction curves were also used to undergo depth sequencing analysis, which was enough to capture microbial diversity. All these methods helped investigate yaks’ community composition in dietary groups (FA, FB, FC, and FD).

The distinctive abundance of microbial species in response to dietary treatments was examined using Linear Discriminant Analysis Effect Size (LEfSe) (Version: 1.0) and STAMP (Version: 2.1.3). The species abundance between groups was performed with the help of STAMP and differences were investigated using the Wilcoxon rank-sum or Kruskal–Wallis test. Additionally, PICRUSt2 was utilized to predict the functional profiles of different microbial communities across the groups based on ASV sequences. Ultimately, the configuration of the microflora was acquired through sequencing to forecast the metabolic functions of the microflora. The Kyoto Encyclopedia of Genes and Genomes (KEGG) and Clusters of Orthologous Groups (COG) databases were used to provide functional pathways about microbial communities, and collectively these methods were focused on providing information about metabolic functions governed by different dietary treatments such as amino acid biosynthesis, immune-related pathways, and different energy metabolisms.

### 2.5. Sequencing Quality and Data Preprocessing

Following the sequencing of 16S rRNA gene (V3–V4 regions), raw paired-end genes were fetched for every single sample. To monitor the quality of the data, Q20 and Q30 scores were used, which provided information about the percentage of bases with sequencing accuracy greater than 96% and 92%, respectively. All the samples that received Q30 values more than 90% were considered high-quality sequencing data suitable for downstream analysis. Clean reads were created after quality assurance and chimera removal using QIIME 2 and DADA 2. Every single sample conserved enough reads, ensuring sufficient sequencing depth for microbial diversity analysis. In addition, the GC content of the sequences averaged 53%, which falls within the anticipated range for microbial DNA, confirming data accuracy for further use.

### 2.6. Statistical Analysis

Arithmetical methods were used to analyze the importance of distinctions in microbial diversity amongst different dietary groups. In the alpha diversity, correlations between groups and indices, such as Shannon, Simpson, Chao1, and observed species, were performed using *t*-tests for issued data. However, Wilcoxon rank-sum tests were operated for the information that did not fall under general expectations. Additionally, to detect multiple groups simultaneously, Tukey’s Honest Significant Difference (HSD) test was performed to examine pairwise variations. Furthermore, for beta diversity investigation, Permutational Multivariate Analysis of Variance (PER-MANOVA) and Analysis of Similarity (Anosim) tests were operated to investigate the statistical importance of alterations regarding microbial communal composition amongst definitive collections. These tests were dependent on distance matrices such as UniFrac distances (unweighted) and Bray–Curtis dissimilarity (weighted).

The Linear Discriminant Analysis Effect Size (LEfSe) assessment was used to examine the abundant taxa across dietary treatments. This method integrated Kruskal–Wallis and Wilcoxon tests with linear discriminant analysis (LDA) to identify key microbial metagenomic biomarkers that differentiated one dietary group from another. LDA scores greater than two were considered significant. For the other statistical approaches, Statistical Analysis of Metagenomic Profiles (STAMP) was performed, which provided effect sizes and *p*-values for differentiation between groups. STAMP and LEfSe provided detailed information and understanding of how microbial taxa behave when certain changes in dietary protein–energy ratios occur in yaks. One-way ANOVA (linear model) with Tukey’s post hoc test was used to evaluate remarkable modifications between individual dietary groupings. All these assessments were performed using R software (R-4.4.2) with appropriate biochemical and microbial data analysis packages. Then, *p*-values were obtained by using permutations. A *p*-value < 0.05 was judged as significant.

## 3. Results

### 3.1. Analysis of Sequencing Data Results

**Figure 1:**
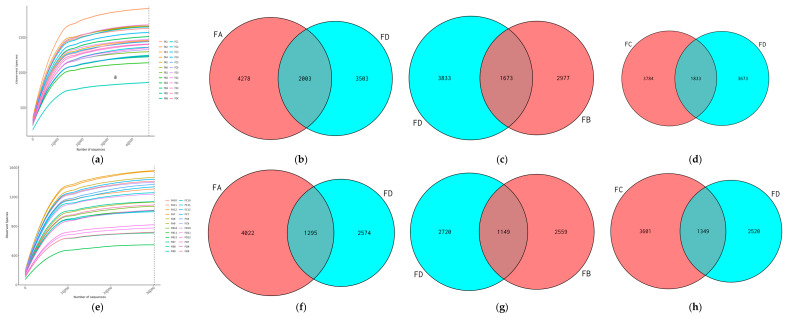
Rarefaction curves and Venn diagrams for samples at day 15 (**a**–**d**) and day 30 (**e**–**h**).

A total of 5,669,645 raw data points (3,189,115 and 2,480,530 from day 15 and day 30, respectively) were obtained from sequencing, with 118,118 ± 2584 (arithmetic mean± SEM) per sample. The ratio of effective sequences to raw sequences was 91.48 ± 0.13 (arithmetic mean ± SEM) per sample (Table 3 and Table 4). As the number of sequences increased, the rarefication curve became flatter, indicating that the potential for an increase in species discovered through sequencing was relatively small, and the sequencing depth already met the analysis needs (Figure 1a,e).

In addition, the gradual flattening of the rarefaction curve with the increase in the number of sequencing data indicates that the depth of sequencing was reasonable and sufficient, covering all the species in the samples under study and fulfilling the prerequisites of further study (Figure 1a,e), confirming the reliably and adequacy of data. In the Venn diagram, the different operational taxonomic units (OTUs) are represented by different colors and the middle represents shared OTUs (Figure 1b–d,f–h).

### 3.2. Microbial Diversity Analysis

#### 3.2.1. Alpha Diversity

**Figure 2:**
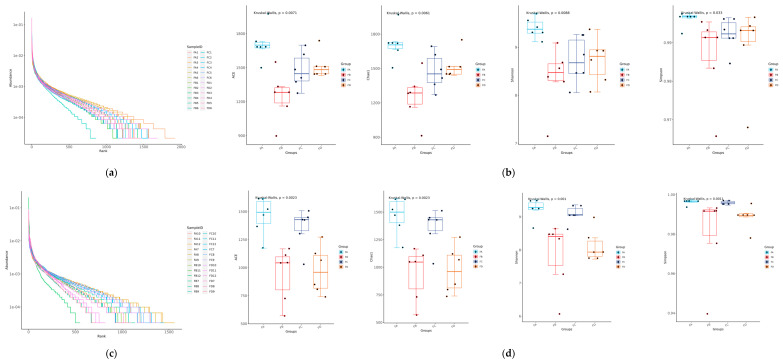
Alpha diversity analysis: rank abundances and other α-diversity indices at day 15 (**a**,**b**) and day 30 (**c**,**d**).

The rank–abundance curve found that among the collected samples, the curves belonging to the FA group had a larger range and smoother shape on the horizontal axis, suggesting that the FA group exhibited higher species abundance and species evenness (Figure 2a,c). The ACE indices, Chao1, Shannon, and Simpson exposed well-defined patterns in yak gut microbiota diversity based on the dietary protein–energy ratios. The FA group gave insights into high species richness and diversity, as indicated by alpha diversity indices (*p* < 0.05). FC represented moderate diversity compared to FA. Meanwhile, the FB and FD groups showed a low level of diversity across all groups, suggesting that the diets of the FA and FC groups might increase gut microbial variety in yaks. The rarity aspect of certain microbial species was revealed in FA and FC, as reflected in the Chao1 and ACE indices (Figure 2b,d).

#### 3.2.2. Beta Diversity

**Figure 3:**
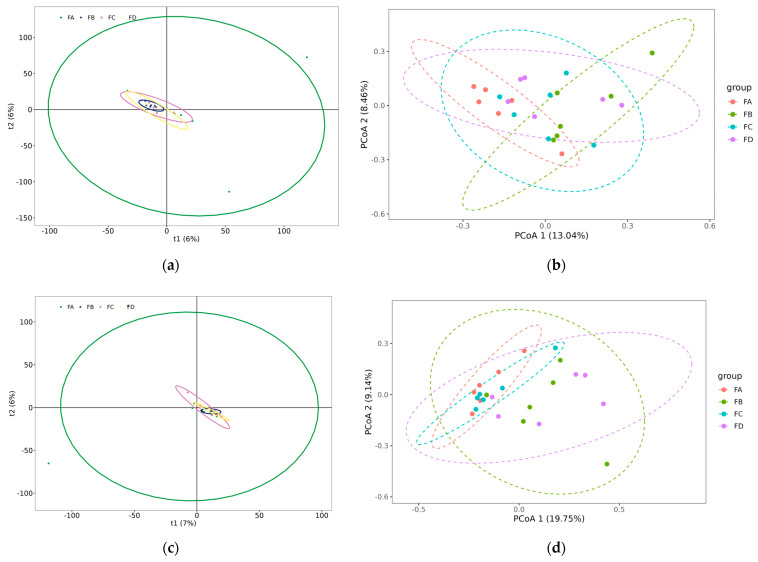
Beta diversity analysis at day 15 (**a**,**b**) and at day 30 (**c**,**d**).

Beta diversity analysis was evaluated to inspect the variations in microbial community configuration amongst certain dietary groups. Principal component analysis (PCA) reduced high-dimensional data to two dimensions, highlighting the most significant differences between samples. Samples with similar microbial compositions appeared closer together on the PCA plot (groups FB, FC, FD). In contrast, the microbial composition similarity between samples in the FA group was lower, and the distance between dots was relatively far (Figure 3a,c). Principal coordinate analysis (PCoA) analyzed the clear clustering of samples according to their dietary groups; results found that changing the ratio of protein and energy in the diet did not significantly impact the differences in gut microbiota composition between groups (Figure 3b,d).

### 3.3. Taxonomic Composition Analysis

The community structure component map showed diverse taxonomical levels of each group. Histograms of the comparative abundance of species were generated, in which the relatively abundant species and their proportions were visually viewed at diverse taxonomic levels.

**Figure 4:**
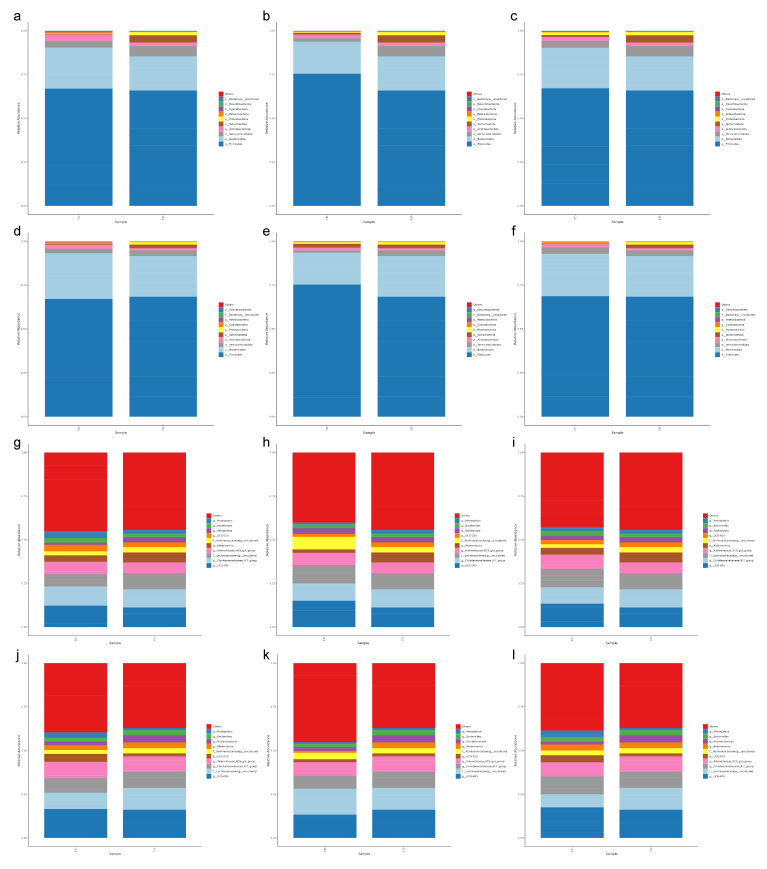
Chart of relative species abundance at the rank of each group in different taxa on day 15 and day 30. This figure shows the top 10 phyla in group EA, EB, and EC (**a**–**c**), and the top 10 genera in group EA, EB, and EC (**d**–**f**) on the 15th day and 10 phyla in group EA, EB and EC (**g**–**i**) and top 10 genera in group EA, EB and EC (**j**–**l**) at 30th day compared to the control group.

At the phylum level, Bacteroidetes, Proteobacteria phyla, and Firmicutes overshadowed the microbial communities across all the groups’ samples. However, their relative abundance varies across different dietary groups. The specific relative abundance was as follows: Firmicutes accounted for the largest proportion (15 d: FA: 66.93%, FB: 75.36%, FC: 67.05%, FD: 65.87%; 30 d: FA: 67.15%, FB: 75.35%, FC: 68.69%, FD: 68.39%); Bacteroidota accounted for the second largest proportion (15 d: FA: 23.35%, FB: 18.34%, FC: 23.19%, FD: 19.43%; 30 d: FA: 25.97%, FB: 18.15%, FC: 24.08%, FD: 23.24%); while Verrucomicrobiota accounted for the third largest proportion (15 d: FA: 3.83%, FB: 1.99%, FC: 3.96%, FD: 5.89%; 30 d: FA: 2.77%, FB: 1.15%, FC: 3.72%, FD: 3.22%). It was found that Firmicutes abundance was much richer in group FB. At the same time, the abundance of Bacteroidota was lower in group FB than in other groups, indicating that the ratio of Firmicutes/ Bacteroidota was higher in group FB (Figure 4a–f). Additionally, *UCG-005* (FA: 12.16%, FB: 15.06%, FC: 13.37%, FD: 11.16%), *Christensenellaceae R-7 group* (FA: 10.95%, FB: 9.83%, FC: 9.33%, FD: 10.37%), and *Rikenellaceae RC9 gut group* (FA: 7.14%, FB: 6.97%, FC: 8.18%, FD: 6.10%) were dominant at genus level in samples collected at the middle of the experiment. The top three genera in samples collected at the end of the experiment were the same as those collected at the middle of the experiment: *UCG-005* (FA: 16.49%, FB: 13.30%, FC: 17.38%, FD: 16.09%), *Christensenellaceae R-7 group* (FA: 8.30%, FB: 7.34%, FC: 10.23%, FD: 9.09%), and *Rikenellaceae RC9 gut group* (FA: 9.30%, FB: 7.86%, FC: 8.02%, FD:8.97%). Results found that the abundance changes in the *UCG-005* and *Christensenellaceae R-7 groups* in the FB group showed an opposite trend compared to other groups. In addition, the relative abundance of the *Rikenellaceae RC9 gut group* increased in groups FA, FB, and FD as the experiment progressed (Figure 4g–l).

**Figure 5:**
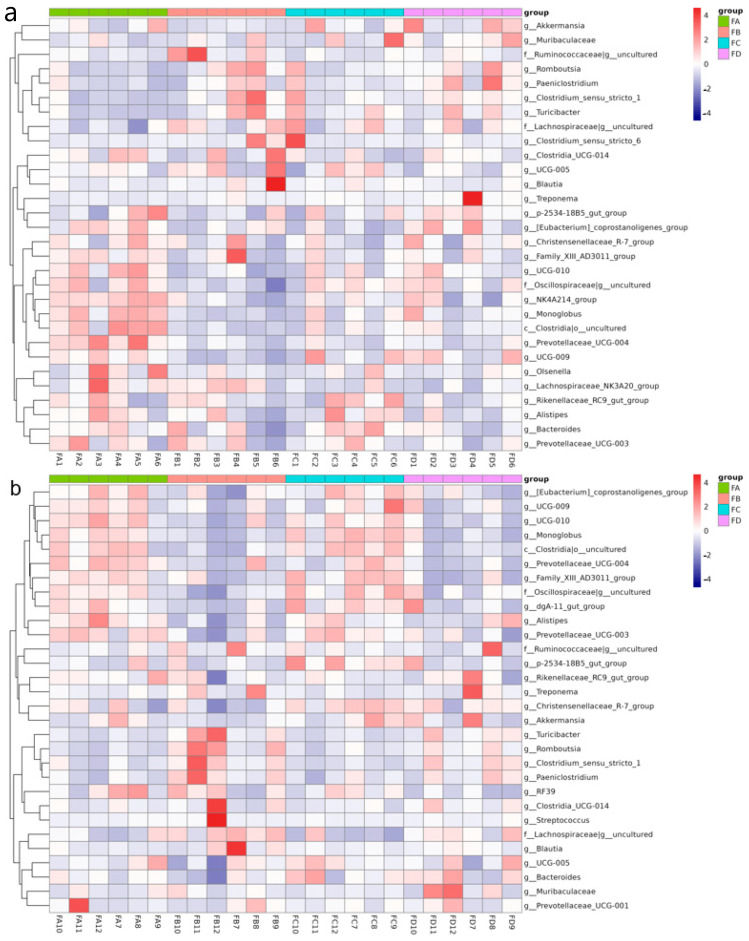
Cluster heat map of species abundance at the level of genus at day 15 (**a**) and day 30 (**b**).

Heatmaps visualize the microbial abundance across samples and groups using color gradients. Heat maps of the top 30 genera in samples collected in the two stages of the experiment are presented in Figure 5a,b.

### 3.4. Analysis of Species Differences Between Groups

**Figure 6:**
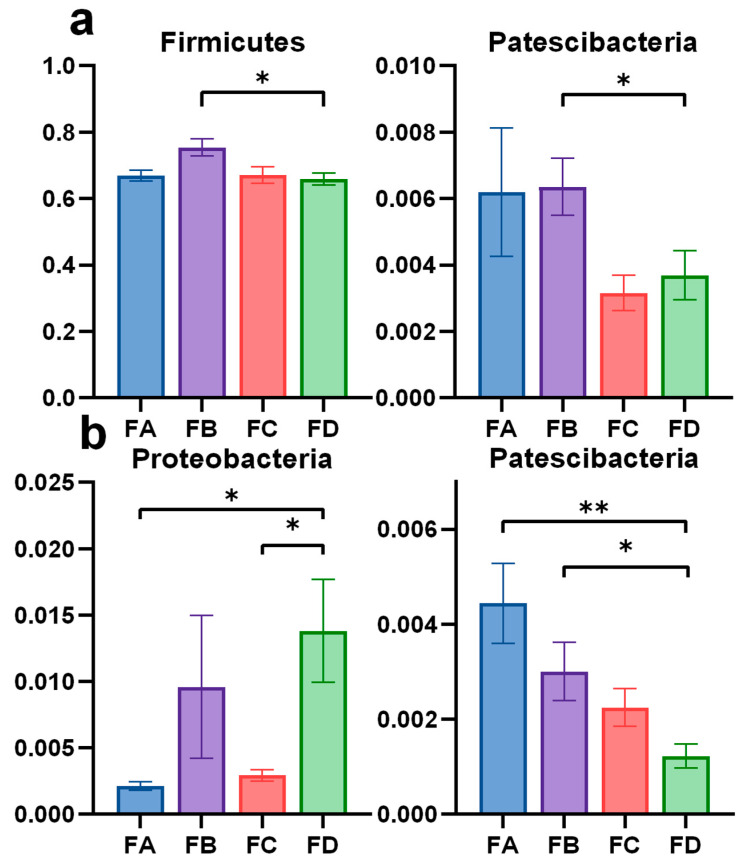
Significance analysis of gut microbiota in different groups at the phylum level at day 15 (**a**) and day 30 (**b**). Data are represented as means ± SD. * *p* < 0.05, ** *p* < 0.01.

In the phylum level, the abundance of Firmicutes and Patescibacteria in group FB was significantly higher than in the control group in samples collected on the 15th day of the experiment (Figure 6a). On the 30th day of the experiment, Proteobacteria abundance was rarer in group FA and FC than in group FD. Simultaneously, Patescibacteria abundance was richer in group FA and FB than in group FD (Figure 6b).

**Figure 7:**
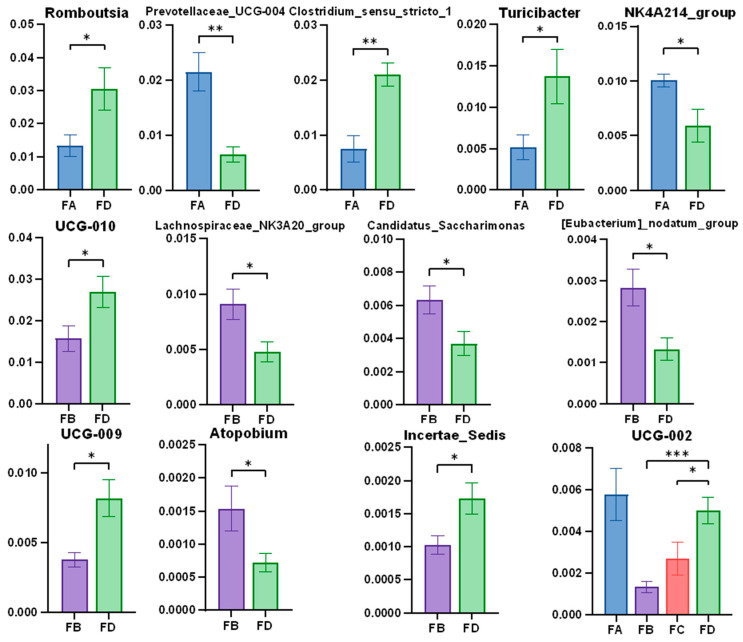
Significance analysis of gut microbiota in different groups at the genus level (15 d). Data are represented as means ± SD. * *p* < 0.05, ** *p* < 0.01, *** *p* < 0.001.

At the genus level, it was found that on the 15th day of the experiment, in group FA, the abundance of *Romboutsia*, *Clostridium sensu stricto 1*, and *Turicibacter* were significantly low. In contrast, the abundance of the *Prevotellaceae UCG-004* and *NK4A214 group* were significantly high compared to group FD. *Lachnospiraceae NK3A20 group*, *[Eubacterium] nodatum group*, *Incertae sedis*, *Atopobium*, and *Candidatus saccharimonas* were richer in group FB while *UCG-010*, *UCG-009*, and *UCG-002* were rare in group FB compared to group FD. In addition, *UCG-002* in group FC was significantly richer than in group FD, as well (Figure 7).

**Figure 8:**
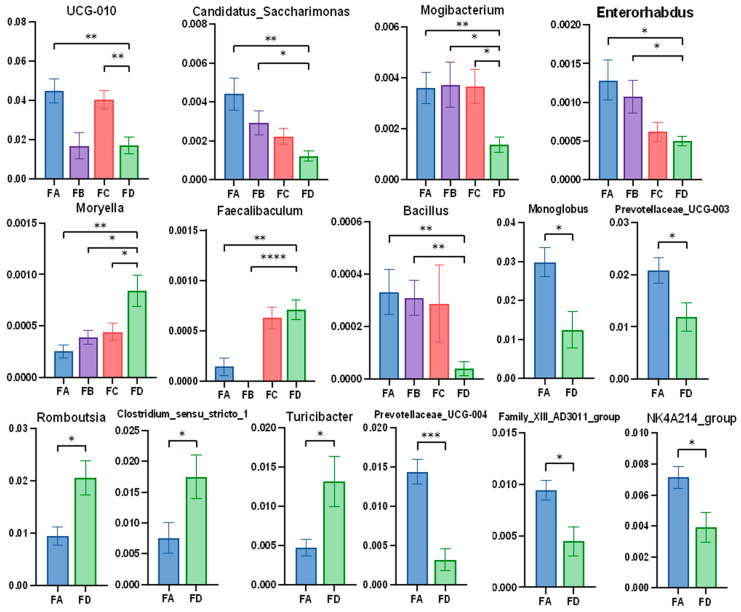
Significance analysis of gut microbiota in different groups at the genus level (30 d). Data are represented as means ± SD. * *p* < 0.05, ** *p* < 0.01, *** *p* < 0.001, **** *p* < 0.0001.

In the samples collected at the end of the experiment, *Mogibacterium* was significantly richer, while *Moryella* was visibly rare in the groups with dietary changes compared to the group of yaks with normal diet. The abundance of *UCG-010*, *Candidatus_saccharimonas*, *Enterorhabdus*, *Bacillus*, *Monoglobus*, *Prevotellaceae, UCG-003/004*, Family *XIII AD3011 group*, and *NK4A214* group were higher in the FA group than the control group. Additionally, the abundance *of Faecalibaculum*, *Romboutsia*, *Clostridium sensu stricto 1*, and *Turicibacter* were lower in group FA than in group FD. It was found that *Candidatus_saccharimonas*, *Enterorhabdus*, and *Bacillus* also had a higher abundance in group FB than FD, while *Faecalibaculum* was invisible in group FB. Additionally, *UCG-010* was enriched in group FC (Figure 8).

**Figure 9:**
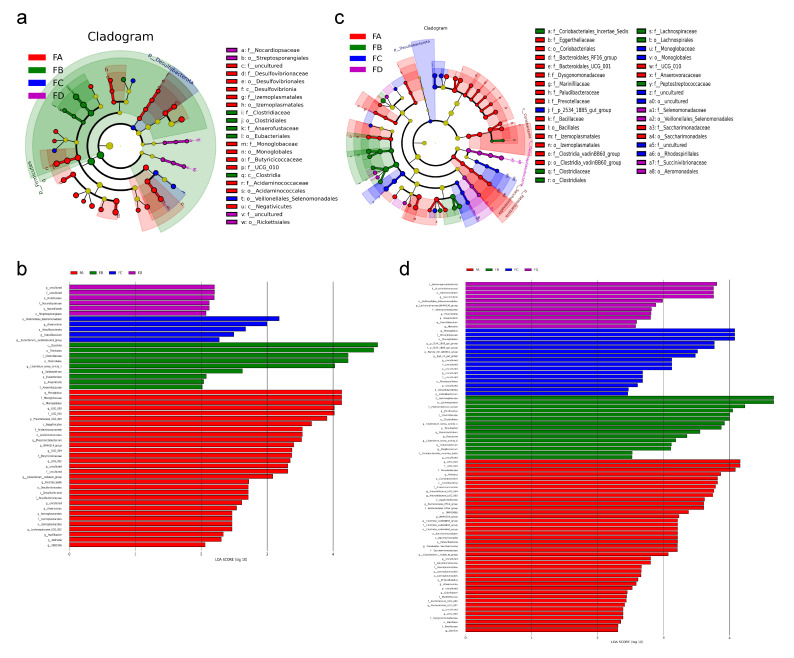
LEfSe analysis of gut microbiota in different groups at day 15 (**a**,**b**) and day 30 (**c**,**d**).

LEfSe analysis, like a *t*-test, revealed the trend of changes in gut microbiota among different groups of yaks. Figure 9a,b show the significantly enriched species in each group of samples collected during the middle of the experiment. Results found that order *Rickettsiales* and some uncultured genera in this order were significantly richer in the control group, as well as genus *Nocardiopsis* (order Streptosporangiales, family Nocardiopsaceae). The abundance of *UCG 010* (family UCG 010), *Monoglobus* (order Monoglobales, family Monoglobaceae), *Prevotellaceae UCG 004*, *UCG 009* (family Butyricicoccaceae), *NK4A214_group*, *UCG 002*, *Phascolarctobacterium* (family Acidaminococcaceae), *Anaerovorax*, *Izemoplasmatales*, *Papillibacter*, *Mailhella*, and so on were higher in group FA. Phylum Firmicutes, class Clostridia, order Clostridiales, Eubacteriales, family Clostridiaceae, Anaerofustaceae, and genera *Clostridium sensu stricto 1*, *Solobacterium*, and *Anaerofustis* were significantly enriched in group FB. Additionally, phylum Desulfobacterota, order Veillonellales Selenomonadales, genus *Anaerovibrio*, *Faecalibaculum*, and *Eubacterium* oxidoreductase group were significantly increased in group FC.

In samples collected at the end of the experiment, multiple genera were found richer in the group of yaks fed with a normal diet, such as *Succinivibrio*, *Moryella*, *Anaerovibrio*, *Faecalibaculum*, *Lachnospiraceae_NK4A136_group*, and *Fournierella*. Compared with other groups, the most diverse species were discovered in the FA group, with 41 at the genus level, including *UCG 010*, *Alistipes*, *Prevotellaceae UCG 003/004*, *Enterorhabdus*, and so on. *Romboutsia*, *Clostridium_sensu_stricto_1*, *Turicibacter*, etc., were discovered to have higher abundance in group FB, while *Monoglobus*, *Family_XIII_AD3011_group*, *Catenibacterium*, and serval uncultured genera were found to be richer in group FC (Figure 9c,d).

### 3.5. Functional Prediction Analysis

**Figure 10:**
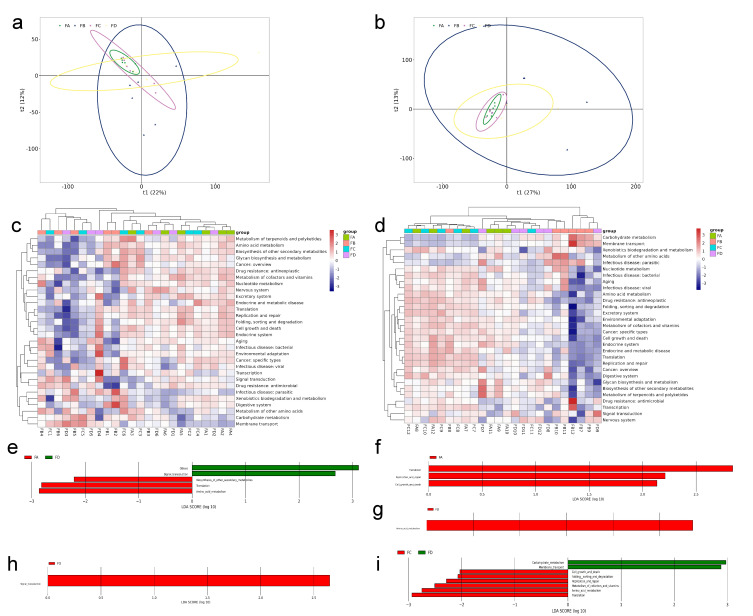
Function analysis of gut microbiota in different groups: (**a**,**b**) PCA analysis; (**c**,**d**) heatmaps; (**e**–**i**) LEfSe analysis.

The PICRUSt2 tool analyzed the efficiency ability of microbial communities, and the analysis was based on KEGG. In KEGG, the functional pathways differ across certain dietary groups, which reflects the influence of protein–energy ratios on microbial metabolism. PCA analysis was conducted to reveal similarities or differences in the overall functions of microflora across different groups. In the PCA plot, closer distances indicate greater similarity in community function among samples. PCA was performed on the predicted KEGG functions, and the results are presented. It can be seen from Figure 10a,b that there was not much difference in microbial community function among the groups. As carried out on the predicted KEGG functions, with the results in Figure 10c,d, the heatmap uses color variations to represent data in 2D matrices or tables, providing an intuitive display of functional abundance values through color shades.

LEfSe analysis further revealed significantly enriched bacterial metabolic pathways in each group. On the 15th day, group FA supported “Amino acid metabolism”, “Translation”, and “Biosynthesis of other secondary metabolites” pathways, while the FD group had more signal transduction metabolism-related pathways compared to the FA (Figure 10e) and FC groups (Figure 10h). On the 30th day, compared to the control group, FA supported pathways of “Translation”, “Replication and repair”, and “Cell growth and death” (Figure 10f); FC supported pathways of “Amino acid metabolism”, “Translation”, “Metabolism of cofactors and vitamins”, “Replication and repair”, “Cell growth and death”, and “Folding sorting and degradation” (Figure 10i). Additionally, the pathway of “Amino acid metabolism” in FD was richer than FB (Figure 10g), and the pathways of “Carbohydrate metabolism” and “Membrane transport” in FD were richer than FC (Figure 10i).

## 4. Discussion

The yak breeding industry is an important economic pillar for the Tibetan people. The reproductive rate of yaks significantly influences the yak breeding industry, and the recovery time of female yaks from estrus postpartum is an important factor in the yak reproductive rate. Dietary supplementation can improve the physical fitness of postpartum weaned yaks and accelerate their recovery, with protein and energy supplementation being the most important [6,36,37]. This study assessed the effects of varying protein–energy ratios in diets on the intestinal microbiota of postpartum weaned yaks. The results demonstrated notable changes in community structure and microbial diversity, along with shifts in functional potential due to dietary adjustments [38].

Furthermore, in the given study, the impacts of dietary protein–energy ratios on microbial diversity were evaluated by alpha and beta diversity. The alpha diversity analysis showed enhanced levels of microbial richness and diversity in groups FA and FC (the diets with high protein ratios), as shown in Shannon and Chao1 indices. This indicated that enough protein supported the diversity in microbial communities. However, the FB group showed lower microbial diversity, showing a potential reduction in gut microbial flexibility. Likewise, previous studies indicated that diverse microbial communities enhance resistance to pathogens and improve nutrient absorption [39]. This suggests that the lower level of microbial diversity in the FB group could negatively affect yak health. Beta diversity analysis confirmed that dietary composition influenced community structures, although not significantly. PCoA plots revealed a clear clustering of samples, indicating that varying protein–energy ratios resulted in distinct microbial profiles. These findings were similar to the reports that indicated that various dietary strategies have led to modifications in the gut microflora of yaks [40].

Firmicutes were high in the FB group, which showed high fiber fermentation under certain dietary conditions [41]. Bacteroidetes were less abundant in the FB group compared to FD, which showed decreased carbohydrate metabolism, indicating lower carbohydrate digestion. Additionally, it was found that the ratio of Firmicutes/ Bacteroidetes was lower in group FA and FC, while it was higher in group FB. Previous studies have suggested that the increase in this ratio is related to obesity, indicating that a high-energy, low-protein diet can cause fat accumulation [42]. Firmicutes degrade fiber and convert cellulose into VFAs, while Bacteroidetes facilitate protein and carbohydrate digestion and absorption and strengthen the enteric immune system [43,44]. SCFAs (propionate, butyrate, acetate) are the major byproduct of gut bacteria. They break carbohydrates that are indigestible and are recognized as vital energy materials; have anti-cancer and anti-inflammatory characteristics; can lower cholesterol and fat storage; regulate the pH of the intestine; and also avoid predatory harmful germs from entering and sticking [45,46]. The F/B ratio is a critical indicator for assessing interrelations between gut microbes and host energy metabolism; a higher F/B ratio is positively correlated with nutrient absorption and fat deposition [47,48]. Firmicutes in the GI tract was found to be positively correlated with energy metabolism [38].

Actinobacteriota increases intestinal homeostasis, carbohydrate breakdown, immune reactions, and inflammation [49]; also, as a probiotic this group prevents a range of resistant pathogenic bacteria [50]. Also, Zhang et al. (2020) found that Actinobacteria, Verrucomicrobia, Proteobacteria, and Patescibacteria were the main phyla in domestic yaks, and a substantial positive relationship between short-chain fatty acids (SCFAs) and Patescobacteria was found [51]. Currently, it is not possible to determine the impact of fat growth on postpartum yaks. Compared with the control group, the abundance of Proteobacteria in FA and FC groups was significantly downregulated, revealing the benefits of a relatively high protein diet on the digestive health of yaks.

Yin et al. found that the addition of additives to the diet significantly improved endothelial function in rats, accompanied by a significant decrease in the relative abundance of *Romboutsia* and *Clostridium-senss_stricto_1* [52]. In the therapeutic effect of salidroside on colitis in mice, it was found that the abundance of *Turicibacter* and *Romboutsia* decreased with the alleviation of intestinal inflammation [53]. In addition, the *NK4A214_group* and *Prevotellace-UCG-003* levels significantly increased in piglets fed with the new probiotics [54]. Changes in the bacterial genera mentioned above were consistent with the trend of gut microbiota in the FA group, suggesting beneficial health effects. *Ruminococcus* was found to be enriched in group FA; a previous study considered that the vitality of *Ruminococcus* was compatible with serum protein and albumin levels in a positive perspective, which suggested that this genus played a key role in nitrogen utilization and protein digestion. This finding aligns with the previous study, which showed that *Ruminococcus* had a key role in the breakdown of fibrous plant material in ruminants *Ruminococcus* [55]. *Lachnospiraceae_NK3A20_group* is one type of cellulose-degrading bacteria that improves the digestion efficiency of yaks and promotes the absorption of nutrients [56], which is enriched in group FB. The decrease in abundance of *Moryella* is positively correlated with the improvement of glucose homeostasis, indicating that the increase in energy and protein in feed improves carbon-, water- and lipid-related metabolism in postpartum weaned yaks [57]. The abundance of *Enterorhabdus*, which was found to be negatively correlated with inflammatory bowel disease (IBD) [58], increased in the experimental groups. In addition, bacteria genera that produce SCFAs were enriched in the FA and FB groups, such as *Candidatus saccharimonas* [59], which decreases in cases of rectal cancer [60]. In summary, the diets (high energy) of FA and FB groups have more advantages in affecting the health of postpartum yaks.

Moreover, the functional exploration between discrete dietary groups explained the metabolic roles of microbial communities that had undergone distinct dietary treatments. Microbial metabolites affect animal intestinal health, as well as the cardiac and brain systems [61]. According to the KEGG pathway, amino acid biosynthesis pathways were enriched in the FA and FC groups, which showed that these diets enhanced microbial growth and protein metabolism, pointing towards efficient protein utilization [62]. The FA and FB groups supported energy metabolism in the pathway because these groups involved amino acid biosynthesis and short-chain fatty acid production. However, this is in addition to the enrichment of metabolic processes involving the utilization of exogenous energy [18]. Actinobacteria’s secondary metabolites, primarily antimicrobial peptides, have a strong probiotic effect, improving immunity [63,64,65]. In contrast, the FD group had more lipid metabolism and stress responses, pointing out potential metabolic imbalances. Additionally, compared with the FC group, the enrichment of carbohydrate metabolic pathways in the FD group provides insights into the significance of carbohydrates as an energy source. Most bacteria in Proteobacteria are related to intestinal dysbiosis and inflammation.

The findings of this study provided beneficial insights into optimizing dietary strategies for yaks in high-altitude environments. Keeping in mind the significance of microbial diversity maintenance for gut health, diets with good levels of protein–energy ratios, such as those in the FA and FC groups, are recommended to support microbial health and optimal microbial functions. Meanwhile, imbalanced diets like those offered to the group FD, compromise animal health over time because of low gut flexibility and increased metabolic stress. These study findings are important because yaks are dependent on microbial fermentation to digest nutrient-poor forage in rigorous environmental conditions [66]. Livestock breeders can enhance nutrient absorption and productivity by properly balancing energy and protein ratios in the diet, which will reduce metabolic stress. Moreover, understanding microbial biomarkers opens a pathway for further research to develop microbiome-target interventions, such as probiotics or dietary supplements, to improve animal health.

## 5. Conclusions

This study highlighted the significant influence of dietary protein–energy ratios on the intestinal microbiota of postpartum weaned yaks. As fed to groups FA and FC, balanced feed with optimal protein and energy proportions demonstrated enhanced microbial diversity and richness and positively influenced metabolic pathways. These results explored the integral role of gut microbiome in the health and productivity of yaks, especially at high altitudes. In contrast, imbalanced diets, as fed to group FD, resulted in gut dysbiosis and metabolic stress. The given study also provided valuable insights into dietary manipulations to enhance nutrient absorption and utilization. The increased relative abundance of beneficial genera like *Ruminococcus* and functional pathways related to amino acid and energy metabolism indicate that protein-rich diets positively influence gut health and metabolic efficiency. These results provide a practical approach to improving yak management in challenging environmental conditions. Future research should explore accurate diet formulations and microbiome-targeted interventions, such as probiotics or prebiotics, to further optimize health and productivity adding longitudinal studies to check the long-term effects of dietary manipulations.

## Figures and Tables

**Table 1:** Nutritional composition of three different formulas.

Ingredient	Formula 1	Formula 2	Formula 3
Alfalfa hay	20.00	20.00	20.00
Oat grass	40.00	30.00	40.00
Corn	16.00	26.50	24.00
Wheat bran	10.00	7.00	10.00
Soybean meal	5.00	5.50	1.00
Cotton meal	5.00	5.50	1.00
Rapeseed oil	0.00	1.50	0.00
4% premix	4.00	4.00	4.00

**Table 2:** Three different groups provided with varying energy and protein ratios.

Nutrient Composition	Group FA	Group FB	Group FC
DM (Dry Matter)	87.90	88.24	87.71
ME (Metabolizable Energy)	8.71	9.75	8.93
CP (Crude Protein)	14.64	14.57	11.85
NDF (Neutral Detergent Fiber)	36.61	31.60	35.69
ADF (Acid Detergent Fiber)	22.60	19.16	21.72
Ca	0.91	0.89	0.90
P	0.67	0.64	0.61

**Table 3:** The bacterial sequencing data of yaks on the 15th day.

Sample-Id	Input	Filtered	Percentage of Input Passed Filter	Denoised	Percentage of Input Denoised	Merged	Non-Chimeric	Percentage of Input Non-Chimeric	Total_ASVs	ASV_Counts
FA1	115,116	110,112	95.65	103,026	89.49	61,448	36,350	31.58	20,442	1813
FA2	113,456	108,954	96.03	101,275	89.26	57,494	36,161	31.87	20,442	2051
FA3	102,975	98,558	95.71	93,788	91.08	63,468	44,178	42.9	20,442	1563
FA4	108,040	103,684	95.97	98,127	90.82	64,121	38,577	35.71	20,442	1720
FA5	96,960	92,770	95.68	86,846	89.57	52,644	36,457	37.6	20,442	1705
FA6	105,502	101,305	96.02	94,717	89.78	58,069	37,857	35.88	20,442	1817
FB1	98,598	94,803	96.15	90,121	91.4	64,125	52,268	53.01	20,442	1716
FB2	108,811	104,337	95.89	98,820	90.82	61,101	34,219	31.45	20,442	1586
FB3	108,570	103,993	95.78	98,065	90.32	60,298	38,946	35.87	20,442	1651
FB4	95,175	91,437	96.07	85,232	89.55	48,166	32,300	33.94	20,442	1740
FB5	117,734	113,105	96.07	109,303	92.84	86,204	74,847	63.57	20,442	1893
FB6	107,200	102,710	95.81	97,366	90.83	63,296	43,906	40.96	20,442	1516
FC1	100,180	95,866	95.69	89,291	89.13	49,632	31,022	30.97	20,442	1681
FC2	111,415	106,365	95.47	99,986	89.74	61,810	34,764	31.2	20,442	1820
FC3	109,250	104,913	96.03	98,385	90.05	58,229	36,481	33.39	20,442	2045
FC4	99,676	95,354	95.66	89,594	89.89	56,080	37,217	37.34	20,442	1697
FC5	93,623	89,462	95.56	83,733	89.44	48,820	30,735	32.83	20,442	1710
FC6	93,304	89,959	96.41	85,708	91.86	62,649	52,959	56.76	20,442	1775
FD1	90,623	86,971	95.97	82,503	91.04	53,857	30,226	33.35	20,442	1373
FD2	87,592	84,105	96.02	80,004	91.34	54,069	41,322	47.18	20,442	1542
FD3	95,068	91,079	95.8	85,047	89.46	50,071	33,809	35.56	20,442	1657
FD4	98,520	94,406	95.82	88,846	90.18	55,364	33,199	33.7	20,442	1725
FD5	117,331	112,773	96.12	105,564	89.97	62,498	40,871	34.83	20,442	1921
FD6	104,186	99,571	95.57	93,795	90.03	55,704	37,098	35.61	20,442	1700

**Table 4:** The bacterial sequencing data of yaks on the 30th day.

Sample-Id	Input	Filtered	Percentage of Input Passed Filter	Denoised	Percentage of Input Denoised	Merged	Non-Chimeric	Percentage of Input Non-Chimeric	Total_ASVs	ASV_Counts
FA7	105,673	101,404	95.96	94,979	89.88	56,301	38,939	36.85	18,746	1886
FA8	96,814	93,226	96.29	87,759	90.65	56,440	41,392	42.75	18,746	1854
FA9	83,856	80,247	95.7	76,412	91.12	55,884	46,768	55.77	18,746	1627
FA10	97,885	93,603	95.63	89,629	91.57	68,394	56,221	57.44	18,746	1762
FA11	96,513	92,707	96.06	87,992	91.17	59,231	43,362	44.93	18,746	1831
FA12	96,418	92,764	96.21	87,504	90.75	58,212	40,146	41.64	18,746	1732
FB7	102,382	98,605	96.31	93,911	91.73	62,350	45,333	44.28	18,746	1667
FB8	88,048	84,540	96.02	80,093	90.97	50,847	38,029	43.19	18,746	1611
FB9	86,685	83,038	95.79	78,615	90.69	53,973	42,589	49.13	18,746	1448
FB10	93,805	89,745	95.67	84,789	90.39	54,445	41,501	44.24	18,746	1657
FB11	75,933	73,065	96.22	68,505	90.22	42,077	32,844	43.25	18,746	1408
FB12	97,497	93,812	96.22	88,996	91.28	56,037	39,333	40.34	18,746	1515
FC7	98,379	94,606	96.16	89,383	90.86	57,079	39,912	40.57	18,746	1506
FC8	104,868	100,971	96.28	95,207	90.79	59,598	41,297	39.38	18,746	1891
FC9	84,018	80,870	96.25	76,352	90.88	50,736	31,912	37.98	18,746	1659
FC10	100,174	96,239	96.07	92,215	92.05	67,542	45,400	45.32	18,746	1707
FC11	76,239	73,279	96.12	69,037	90.55	45,075	25,968	34.06	18,746	1515
FC12	111,373	107,334	96.37	102,331	91.88	73,932	41,885	37.61	18,746	1717
FD7	101,140	97,224	96.13	92,285	91.24	63,675	43,238	42.75	18,746	1611
FD8	97,161	93,613	96.35	88,736	91.33	60,260	38,991	40.13	18,746	1755
FD9	107,791	103,838	96.33	99,104	91.94	71,510	39,277	36.44	18,746	1458
FD10	102,844	99,007	96.27	94,298	91.69	66,928	41,241	40.1	18,746	1598
FD11	110,069	106,145	96.43	100,825	91.6	66,725	41,017	37.26	18,746	1846
FD12	92,541	88,930	96.1	84,522	91.33	55,260	37,473	40.49	18,746	1391

## Data Availability

The raw data of yaks in this study were deposited in the NCBI database under accession number PRJNA1185163, https://www.ncbi.nlm.nih.gov/search/all/?term=PRJNA1185163 (accessed on 12 November 2024).
